# Supplementary material for: Acceptance and compliance with micronutrient powder (MNP) among children aged 6–23 months in northern Nigeria
Source: PLOS Glob Public Health. 2022 Oct 17;2(10):e0000961. doi: 10.1371/journal.pgph.0000961 (PMC10022258; doi:10.1371/journal.pgph.0000961)
Supplement: S1 Dataset — (ZIP) [file pgph.0000961.s008.zip › S8_Dataset/Direct Observations.zip!/Direct Observations/010705_A_SO_AD_2_DO.pdf]

### 1.1.1 Instrument F –Direct Observation Form

| |
|---|
| **<<Instrument F Direct Observation Form>>** |
| **PI Name:** PragyaMathema,Stephen Kodish<br>**Study Title:** Formative research to develop an infant and young child feeding (IYCF) intervention with micronutrient powder (MNP) for children aged 6 – 23 months<br>**PI Version/Date:** Version1.0/January 24, 2015 |

Date: _July 01, 2015_____________Location: _Bongo community, LGA -Song

Data Collector: Aisha Aliyu Dahiru________ Participant ID: __

| |
|---|
| Main goals of this direct observation are to answer the following questions:<br><br>1. In this household, what would be the best way(s) to give nutritional supplements to young children? Why?<br><br>2. In this household, describe the food sharing that you observe throughout the meal. Are there any predictable patterns?<br><br>3. In this household, who is favored and how is this favoritism expressed during meal preparation and eating? |

(TEAR OFF AT PERFORATED DOTTED LINE BELOW)

------------------------------------------------------------------------------------------------------------------------

PARTICIPANT ID: __

Household Composition Table (Fill out as you do data collection)

| INITIALS | SEX (M/F) | AGE (estimated) | Relationship to household | Description |
|---|---|---|---|---|
| M | F | 24YRS | Mother | Wearing purple dress. |
| B | M | 11 MONTHS | Baby boy (son) | Wearing green trouser and shirt |
| N1 | F | 24 YRS | Neighbor | Wearing brown head gear with blue wrapper |
| H | M | 32 YRS | Husband | Wearing white shirt with black trouser |
| N2 | M | 34 YRS | Husband's friend | wearing white shirt black trouser |
| N3 | F | 32 YRS | Husband friend | Wearing brown dress |
| | | | | |
| | | | | |

| | | | | |
|---|---|---|---|---|
| | | | | |
| | | | | |
| | | | | |
| | | | | |
| | | | | |
| | | | | |
| | | | | |

PARTICIPANT ID: __________

| TIME | ACTOR INITIALS | RECIPIENT INITIALS | WHAT HAPPENED | FOOD | Reactivity |
|---|---|---|---|---|---|
| 8.30 | M | | Sweeping | | |
| 8:35 | M | | Sweeping | | |
| 8:40 | M | | Sweeping | | |
| 8:45 | M | | Sweeping | | |
| 8:50 | M | | Washing utensils | | |
| 8:55 | B | | Crying | | |
| 8:55 | M | B | Carrying baby on her back | | |
| 9:00 | M | | Washing utensils | | |
| 9:05 | M | | Washing utensils | | |
| 9:10 | M | | Starting fire | | |
| 9:15 | M | | Starting fire to boil water | Water | |
| 9:20 | M | | Mixing akamu in a bowl | Akamu | |
| 9:25 | M | | Adding boiling water into akamu and stirring | | |
| 9:30 | M | | Still adding boiling water into bowl of akamu and stirring | Water, akamu | |
| 9:35 | M | | Akamu is ready to be served | Akamu | |
| 9:35 | M | | Serving akamu into a cup and letting it to cool | Akamu | |
| 9:40 | M | | Adding MNP into cup of akamu, | Akamu | |

| TIME | ACTOR INITIALS | RECIPIENT INITIALS | WHAT HAPPENED | FOOD | Reactivity |
|---|---|---|---|---|---|
| | | | stirring to mix well. The MNP easily dissolved in the akamu. The mother used a finger to taste if the akamu is cold enough to feed the baby | | |
| 9:45 | M | B | Feeding the baby the akamu mixed with MNP | Akamu | |
| 9:46 | M | B | Feeding the baby with a spoon. The baby is anxiously taking the akamu | Akamu | |
| 9:47 | M | B | Feeding the baby | Akamu | |
| 9:48 | M | B | Feeding the baby | Akamu | |
| 9:49 | M | B | The baby refused to open mouth to take the akamu. The mother is trying to forcefully feed the baby but he still refused to open his mouth. | | |
| 10:00 | M | B | The mother gives up and wash the baby's mouth . keeping the akamu cup aside coverd. | | |
| 10:05 | M | B | Bathing the baby | | |
| 10:10 | M | B | Bathing the baby | | |
| 10:15 | M | B | Dressing the baby | | |
| 10:20 | B | | Playing on the mat under mango tree. | | |
| 10:25 | M | | Washing clothes | | |
| 10:30 | M | | Washing clothes | | |
| 10:30 | B | | Playing with sand on the mat | | |
| 10:35 | M | | Spreading out clothes to dry on the rope | | |
| 10:40 | M | B | Breastfeeding the baby carrying the baby on her laps | Breast milk | |
| 10:45 | M | B | Breastfeeding the baby | Breast milk | |
| 10:50 | M | B | Breastfeeding the baby | Breast milk | |
| 10:55 | B | | The child is sleeping still breastfeeding | Breast milk | |
| 11:00 | M | B | Lays down baby and lay down | | |

| TIME | ACTOR INITIALS | RECIPIENT INITIALS | WHAT HAPPENED | FOOD | Reactivity |
|---|---|---|---|---|---|
| | | | beside him | | |
| 11:00 | B | | Sleeping beside mother | | |
| 11:05 | M | | Sleeping beside the child | | |
| 11:10 | B | | Sleeping | | |
| 11:15 | B | | Sleeping | | |
| 11:20 | B | | Sleeping | | |
| 11:25 | M | | Sleeping beside the  child | | |
| 11:30 | B | | Sleeping | | |
| 11:35 | B | | Sleeping | | |
| 11:40 | B | | Sleeping | | |
| 11:45 | M | | Sleeping beside the child | | |
| 11:50 | B | | Sleeping | | |
| 11:55 | B | | Sleeping | | |
| 12:00 | B | | Sleeping | | |
| 12:05 | B | | Crying | | |
| 12:05 | M | | Carrying the baby | | |
| 12:10 | M | | Washing hands | | |
| 12:10 | M | | Serving akamu from the flask | Akamu | |
| 12:15 | M | | Stirring akamu to cool | Akamu | |
| 12:15 | B | | Sitting beside mother crying | | |
| 12:20 | M | B | Carrying baby on her laps . give the child akamu with the cup. The baby is drinking the akamu with ease | Akamu | |
| 12:21 | M | B | Giving the baby akamu with ease | | |
| 12:22 | M | B | Feeding child | | |
| 12:23 | M | B | Feeding the child | Akamu | |
| 12:24 | M | B | Feeding | Akamu | |
| 12:25 | M | B | Washing the child's hands and mouth after giving him the akamu | | |
| 12:30 | M | B | Changing the baby's cloth | | |
| 12:35 | M | N1 | Chatting with neighbor | | |

| TIME | ACTOR INITIALS | RECIPIENT INITIALS | WHAT HAPPENED | FOOD | Reactivity |
|---|---|---|---|---|---|
| 12:40 | B | | Playing on the mothers | water | |
| 12:45 | M | N1 | Chatting with neighbor | | |
| 12:50 | B | | Lying down beside mother | | |
| 12:55 | M | N1 | Chatting with neighbor | | |
| 1:00 | B | | Sleeping beside the mother | | |
| 1:00 | M | | Lying down beside the baby | | |
| 1:10 | B | | Sleeping | | |
| 1:15 | M | | Sleeping beside the baby | | |
| 1:20 | B | | sleeping | | |
| 1:25 | M | | Sleeping beside the baby | | |
| 1:30 | B | | Sleeping | | |
| 1:35 | B | | Sleeping | | |
| 1:40 | B | | Sleeping | | |
| 1:40 | B | | Sleeping | | |
| 1:45 | B | | Sleeping | | |
| 1:50 | M | | Sleeping with baby | | |
| 1:55 | H | N2,N3 | Comes in chatting with friends | | |
| 1:55 | M | | Offering husband friends drinking water | Water | |
| 2:00 | H | M,N2,N3 | Chatting with mother and friends | | |
| 2:05 | M | B | Carrying baby on her lap and consoling the baby | | |
| 2:10 | M | B | Breastfeeding the child | | Breast milk |

| TIME | ACTOR INITIALS | RECIPIENT INITIALS | WHAT HAPPENED | FOOD | Reactivity |
|---|---|---|---|---|---|
| 2:15 | M | B | Breastfeeding the child | Breast milk | |
| 2:20 | M | B | Breast feeding the child | Breast milk | |
| 2:25 | M | B | Carrying child on her lap playing with the child | | |
| 2:30 | M | B | Lying down with the child on the foam in the sitting room | | |
| 2;35 | B | | Lying beside mother | | |
| 2:40 | B | | Lying down quietly beside mother | | |
| 2:45 | H | B | Carrying baby on his laps | | |
| 2:50 | H | B | Playing with baby | | |
| 2:55 | M | | Sitting on the verandah | | |
| 2:55 | H | B | Playing with the baby in the sitting room | | |
| 3:00 | M | B | Carrying child | Breast milk | |
| 3:00 | M | B | Washing the child | | |
| 3:00 | H | | Washing off the stool from his trouser | | |
| 3:05 | M | B | Dressing the baby | | |
| 3:10 | M | B | Carrying the child on her back | | |
| 3:15 | M | B | Carrying baby on her back | | |
| 3:20 | H | M | Chatting with the mother | | |
| 3:25 | H | M | Chatting with mother | | |
| 3:30 | M | H | Serving food in a bowl for the household | Jollof rice | |
| 3:30 | H | | Washing his hands | | |
| 3:30 | M | B | Washing the baby hands | | |

| TIME | ACTOR INITIALS | RECIPIENT INITIALS | WHAT HAPPENED | FOOD | Reactivity |
|---|---|---|---|---|---|
| 3;31 | H | B | Assisting the child to eat. The child is eating from the fathers bowl | Jollof rice | |
| 3:33 | M | B | Assisting the child to eat but he refused to take food from his mother | Jollof rice | |
| 3:34 | H | B | Assisting the child to eat. The child is taking the food from the husband without resistance | Jollof rice | |
| 3:35 | H | B | Still assisting the child to eat while eating | Jollof rice | |
| 3:35 | M | | Eating from a separate bowl alone beside husband and child | Jollof rice | |
| 3:36 | M | | Eating from a separate bowl | Jollof rice | |
| 3:37 | H | B | Still  assisting the child to eat from his bowl | Jollof rice | |
| 3:37 | B | | Trying  to et by himself from the fathers bowl | Jollof rice | |
| 3:38 | B | | Trying to eat by himself from the fathers bowl. Eats the rice one grain at a time | Jollof rice | |
| 3:39 | H | B | Assisting the child to eat from his bowl | | |
| 3:40 | M | B | Washing the baby hands and month with soap and water | | |
| 3:40 | H | | Washing his hands with soap and water | | |
| 3;40 | M | B | Assisting the child to drink water from a large cup | Water | |
| 3:40 | H | | Drinking water from the large cup that the child and mother drank from | Water | |
| 3:45 | B | | Playing beside the mother on the verandah | | |
| 3:45 | B | | Playing beside the mother | | |
| 3:50 | B | | Playing beside the mother | | |
| 3:55 | B | | Crying | | |
| 3:55 | M | B | Carrying the child on her lap | | |

| TIME | ACTOR INITIALS | RECIPIENT INITIALS | WHAT HAPPENED | FOOD | Reactivity |
|---|---|---|---|---|---|
| | | | | | |
| 4:00 | M | B | Playing with the child | | |
| 4:05 | H | M | Chatting with mother | | |
| 4:10 | H | B | Playing with child while the child is on the mothers lap | | |
| 4:15 | M | | Starting the fire | | |
| 4:20 | M | | Washing the utensils | | |
| 4:25 | H | | Playing with the child | | |
| 4:30 | M | | Checking the firewood | | |
| 4:35 | M | | Adding dried vegetable to the food on fire to make soup | Sesame leaves | |
| 4:40 | M | | Soup is ready to be served | Sesame leave soup | |
| 4:45 | M | | Playing with the baby on the verandan | | |
| 4:50 | M | | Adding maize flour to the boiling water to make a meal | Water , maize flour | |
| 4:55 | M | | Meal is ready to be served | Maize meal | |
| 4:55 | M | | Serving meal into a single dish | | |
| 5:00` | M | | Washing hands after making meal | | |
| 5:05 | M | B | Bathing baby | | |
| 5:10 | M | B | BATHING THE BABY | | |
| 5:15 | M | B | Dressing the baby | | |
| 5:20 | M | B | Breast feeding the baby | Breast milk | |
| 5:25 | M | B | Breastfeeding the baby | Breast milk | |
| 5:30 | M | B | Breastfeeding the baby | Breast milk | |
| 5:35 | M | B | Carrying baby on her back | | |
| 5:40 | M | | Washing her hands | | |
| 5:45 | M | | Serving food in a bowl | Maize | |

| TIME | ACTOR INITIALS | RECIPIENT INITIALS | WHAT HAPPENED | FOOD | Reactivity |
|---|---|---|---|---|---|
| | | | | meal | |
| 5:46 | M | | Eating together with baby | Maize meal, sesame leave soup | |
| 5:47 | M | B | Eating meal together with the baby. Feeding the baby | Maize meal, sesame leave soup | |
| 5:48 | M | B | Feeding the baby from her bowl while the child is on her lap | Maize meal, sesame leave soup | |
| 5:49 | M | B | Feeding the baby | Maize meal, sesame leave soup | |
| 5:50 | M | B | Mother giving the child water to drink | Water | |
| 5:55 | M | B | Washing her hands as well as the baby hands | | |
| 5:55 | M | B | Carrying baby on her back | | |
| 6:00 | M | B | Carrying baby on her back | | |
| 6:05 | B | | Baby is Sleeping on the mother back | | |
| | | | | | |
| | | | | | |
| | | | | | |
| | | | | | |
| | | | | | |
| | | | | | |
| | | | | | |
| | | | | | |
| | | | | | |
| | | | | | |
| | | | | | |
| | | | | | |

| TIME | ACTOR INITIALS | RECIPIENT INITIALS | WHAT HAPPENED | FOOD | Reactivity |
|---|---|---|---|---|---|
| | | | | | |
| | | | | | |
| | | | | | |
| | | | | | |
| | | | | | |
| | | | | | |
| | | | | | |
| | | | | | |
| | | | | | |
| | | | | | |
| | | | | | |
| | | | | | |
| | | | | | |
| | | | | | |
| | | | | | |
| | | | | | |
| | | | | | |
| | | | | | |
| | | | | | |
| | | | | | |
| | | | | | |
| | | | | | |
| | | | | | |
| | | | | | |
| | | | | | |
| | | | | | |
| | | | | | |
| | | | | | |
| | | | | | |
| | | | | | |

| TIME | ACTOR INITIALS | RECIPIENT INITIALS | WHAT HAPPENED | FOOD | Reactivity |
|---|---|---|---|---|---|
| | | | | | |
| | | | | | |
| | | | | | |
| | | | | | |
| | | | | | |
| | | | | | |
| | | | | | |
| | | | | | |
| | | | | | |

PARTICIPANT ID: __thatch house with zinc door _______

Primary Questions (Complete <u>after</u> you complete data collection):

From what you have seen observing THIS HOUSEHOLD, please answer the following questions in detail:

| Question | Write your <u>detailed</u> response here |
|---|---|
| 1. In this household, what would be the best way(s) to give nutritional supplements such as an MNP to young children to ensure compliance?<br><br>Why? | The best way to ensure compliance is to encourage the mother to give the child MNP separately before any actual meal because the child did not finish the akamu that was mixed with the MNp. The quantity of the akamu was too much. |
| 2. In this household, who is favored during meal preparation or feeding?<br><br>How is this favoritism | The meal prepared was in a solid state because the parents were favored. The baby was fed by the mother and the father. |

| | |
|---|---|
| expressed? | |
| 3. How could we promote the MNP supplement for this family that would attract their attention?<br><br>Who would they listen to? | MNP can be promoted to this household through the mother.<br>The husband can also serve as influence in promoting the MNP.<br>The household will listen to the community leader, religious leader and also the health workers because they are respected and will not let anyone introduce something that will harm their community members. |
| 4. In this household, what would be the best ways to promote the MNP but limit how much it would be shared?<br><br>Why? | The mother understands the MNP is meant for the baby only but it can be emphasized through the husband because his decisions in the household are final. |
| 5. What other issues and concerns did you see with this family that are important for us to consider with regard to introduction of an MNP?<br><br>Why? | The food prepared was solid. The child did not have a separate bowl when he ate food.<br>The mother was in charge of feeding the baby at one time and the father was in charge at other times. |
| 6. Based on your experience observing this child today, do | There was no reactivity during feeding. |

| | |
|---|---|
| you believe reactivity negatively impacted your ability to see the food-related behaviors of the child?<br><br>Please explain your answer in detail. | |
